# The genetic epidemiology of dementia in under‐represented ethnic communities using studies of the COSMIC Consortium

**DOI:** 10.1002/alz.087350

**Published:** 2025-01-09

**Authors:** Karen A Mather, Anbupalam Thalamuthu, Darren M. Lipnicki, Perminder S. Sachdev, COSMIC Consortium

**Affiliations:** ^1^ Centre for Healthy Brain Ageing (CHeBA), University of New South Wales, UNSW Sydney, NSW Australia; ^2^ Centre for Healthy Brain Ageing (CHeBA), UNSW Sydney, Sydney, NSW Australia; ^3^ Centre for Healthy Brain Ageing (CHeBA), University of New South Wales (UNSW) Sydney, Sydney, NSW Australia

## Abstract

**Background:**

Most human genetic association studies have been undertaken in populations of European ancestry, despite >75% of the world’s population being of Asian or African ancestry. In addition, many of the non‐white genetic studies have had small sample sizes and lack replication. To date, >80 different genetic variants have been identified for late‐onset Alzheimer’s disease (AD) using populations mainly of European ancestry and from high income countries, despite more than ∼60% of dementia cases living in low and middle‐income countries. There is a need to evaluate genetic risk of dementia and related phenotypes in under‐represented populations, such as the multi‐ethnic cohorts of the Cohort Studies of Memory in an International Consortium (COSMIC). Polygenic risk scores (PRS) summarise the known genetic risk for a particular disorder or trait, such as dementia. But the applicability and generalisability of dementia PRSs based on genetic studies of European ancestry is unclear. This study aims to examine the utility and performance of dementia polygenic risk scores (PRSs) derived from European, non‐European, and trans‐ethnic samples for dementia diagnosis and its associations with cognitive impairment in COSMIC studies.

**Method:**

COSMIC participants (middle‐aged to older adults) with genome‐wide genotyping, cognitive and dementia data will be included. Genotypes will be imputed to the appropriate reference panel/s. PRSs will be derived using the appropriate GWAS summary statistics based on European and non‐European based cohorts and trans‐ethnic analyses for the two most common types of dementia (Alzheimer’s disease [AD], vascular dementia [VaD]). Association analyses for the phenotypes of AD, VaD, and cognitive performance will be undertaken using regression adjusting for age, sex, and any other specific covariates.

**Result:**

There are at least 14 COSMIC cohorts with genotyping and dementia/cognitive data available who have recruited participants of European and non‐European ancestry (n>15K), including studies from Japan, South America, Africa, India, and China. Performance of PRSes computed under various models for predicting dementia and cognitive impairment will be presented.

**Conclusion:**

This work will elucidate the utility of specific and multi‐ethnic dementia PRSs predicting the risk of dementia and its endophenotype, cognitive impairment.

